# Dosimetric impact of range uncertainty in passive scattering proton therapy

**DOI:** 10.1002/acm2.13179

**Published:** 2021-04-02

**Authors:** Ruirui Liu, Baozhou Sun, Tiezhi Zhang, Jeffery F. Williamson, Joseph A. O’Sullivan, Tianyu Zhao

**Affiliations:** ^1^ Department of Radiation Oncology Washington University School of Medicine St. Louis MO USA; ^2^ Department of Electrical and Systems Engineering Washington University St. Louis MO USA

**Keywords:** dose calculation, passive scatter proton therapy, proton range uncertainty, retrospective study

## Abstract

**Purpose:**

The objective of this study was to investigate the dosimetric impact of range uncertainty in a large cohort of patients receiving passive scatter proton therapy.

**Methods:**

A cohort of 120 patients were reviewed in this study retrospectively, of which 61 were brain, 39 lung, and 20 prostate patients. Range uncertainties of ±3.5% (overshooting and undershooting by 3.5%, respectively) were added and recalculated on the original plans, which had been planned according to our clinical planning protocol while keeping beamlines, apertures, compensators, and dose grids intact. Changes in the coverage on CTV and DVH for critical organs were compared and analyzed. Correlation between dose change and minimal distance between CTV and critical organs were also investigated.

**Results:**

Although CTV coverages and maximum dose to critical organs were largely maintained for most brain patients, large variations over 5% were still observed sporadically. Critical organs, such as brainstem and chiasm, could still be affected by range uncertainty at 4 cm away from CTV. Coverage and OARs in lung and prostate patients were less likely to be affected by range uncertainty with very few exceptions.

**Conclusion:**

The margin recipe in modern TPS leads to clinically acceptable OAR doses in the presence of range uncertainties. However, range uncertainties still pose a noticeable challenge for small but critical serial organs near tumors, and occasionally for large parallel organs that are located distal to incident proton beams.

## 1 INTRODUCTION

In principle, proton radiotherapy (RT) is capable of superior organs‐at‐risk (OAR) sparing than photon RT due to the Bragg peak lack of exit dose beyond the particle range. These dosimetric advantages are expected to reduce short and long‐term complications^1^, ^2^, ^3^, ^4^, ^5^, ^6^ while maintaining tumor control probability (TCP) for properly selected patients, especially pediatric patients. Precise estimate of proton range at the phase of treatment planning plays a critical key in unlocking the full potential of proton radiotherapy.

The current clinical standard of practice requires a computed tomography (CT) scan at a fixed x‐ray tube potential for the purposes of virtual simulation and treatment planning. Stopping power (SP), defined as proton energy loss per unit distance traveled, is thus estimated from Hounsfield units (HU) in the single‐energy CT (SECT) scan via the well‐established stoichiometric calibration technique.^7^ The accuracy of the estimated proton SP in the current practice is degraded by a variety of factors, including CT acquisition parameters,^8^ imaging noise^9^, ^10^ and artifacts,^11^, ^12^ the calibration methodology,^8^, ^13^ CT grid size,^9^, ^14^ and mean excitation energy (I‐values) in tissues.^15^, ^16^, ^17^, ^18^, ^19^ A major, if not dominant, contribution to this uncertainty (ranging from 1.6% to 5.0% in different tissue groups^20^) is degeneracy of HU numbers in the presence of tissue composition variations, which fails to accurately represent the dependence of I values and SP on tissue composition. These uncertainties associated with SECT proton SP mapping translate to range uncertainties directly. To compensate for range uncertainties, a safety margins of ±3.5% is added to the distal clinical target volume (CTV) boundary to ensure CTV coverage. This procedure has been widely adopted as a standard of practice.^19^, ^21^, ^22^

Without a reliable way of reconstructing proton stopping power ratio (SPR), the proton range estimated from a CT dataset could either under‐cover tumor or overshoot into critical organs that are distal to the tumor. The current clinical standard of practice adds a safety margin, regardless of the actual proton SPR is higher or lower than the true value, maximizing tumor coverage at the cost of possibly excessive toxicity to normal tissues.

The situation could get worse in the actual RT treatment. The approved treatment plan with the added distal margin only represents what the dose distribution would be calculated with the CT calibration curve at the time of plan review and approval. The added distal margin does not eliminate range uncertainties. The actual tumor coverage and sparing of normal tissues approved by radiation oncologist could still deviate significantly from what are achieved in RT treatment.

Although many studies have investigated range uncertainties in proton therapy,^18^, ^22^, ^23^, ^24^ few have evaluated the dosimetric impact of range uncertainties in the true dose actually delivered to patients with clinical plans following the current standard of practice. The objective of this study was to investigate the consequences of range uncertainty on delivered dose distributions for a cohort of patients treated with passively scattered proton therapy.

## 2 MATERIALS AND METHODS

### 2.A Subjects

A cohort of 120 patients treated for brain tumor, lung cancer and low‐risk prostate cancer without pelvic lymph nodes involving between 2013 and 2016 at Washington University’s Kling proton center were selected for this study. Of the 120 patients, the brain tumor, lung tumor, and prostate tumor cases are 61, 39, and 20, respectively. All patients were intended to receive proton irradiation for the full RT course. However, some received IMRT as backup plans due to unexpected machine downtime during their RT courses. Those IMRT backup treatment fractions were ignored in the study. For all patients, we assumed that a full proton RT course were delivered. The fractionation of proton therapy for intracranial malignancy ranged from 28 fractions to 33 fractions, customized on the diagnosis and patient condition. The fractionation is 30 × 2 Gy for lung and 44 × 1.8 Gy for prostate, and both are standard.

### 2.B Treatment planning

All patients were treated with a passive scattering proton machine (Mevion S250, Littleton, MA) and planned in Eclipse V11 (before July 2015) and Eclipse V13(after July 2015). Pencil beam algorithm was used for dose calculation. Despite the variations in versions of treatment planning system, identical beam models were employed for all cases. The difference in dose calculated by the various versions of treatment planning system was <1 cGy and was deemed negligible. All treatments were planned with 3.5% safety margin and approved by board‐certified radiation oncologists. All radiation oncologists treating patients with proton therapy participated in an internal compliance training program for initial and maintenance credentialing. Range uncertainties of ±3.5% were simulated for each beam. Specifically, the shifting proton range was achieved by increasing/decreasing the beam energy to add or subtract 3.5% of the proton range. The shifting range in both directions (overshooting and undershooting by 3.5%, respectively) were added on the original plans and the plans then were recalculated, giving rise to two additional dose distributions with all beams undershooting and overshooting, respectively. All other beam parameters, for example, beamlines, apertures, compensators, dose grids, and monitor units, remained unchanged.

### 2.C Dose analysis

For each site‐specific cohort, appropriate OARs and dose indices were selected as surrogates for treatment toxicities in all three scenarios (original plan, +3.5%, −3.5%), regardless of patient‐specific variations in their location relative to the CTV. Dosimetric endpoints included maximum dose to brainstem, chiasm, left and right optic nerves, for brain tumor patients; V5 Gy and V30 Gy for both esophagus and heart; V20Gy for lung cancer patients; and V40 Gy and V65 Gy for both bladder and rectum for prostate cancer patients. For brain tumor cases, the minimum CTV dose and CTV D95 were also calculated. The CTV V100% dose metric was also reported for lung and prostate cohorts. Differences in dosimetric metrics among the three scenarios were visualized via curves side by side. Boxplots were used to visualize distribution of dose deviations from the original plans, highlighting the median, 25th, and 75th percentiles of the data distribution.

## 3 RESULTS

### 3.A Brain tumor cases

**Fig. 1:**
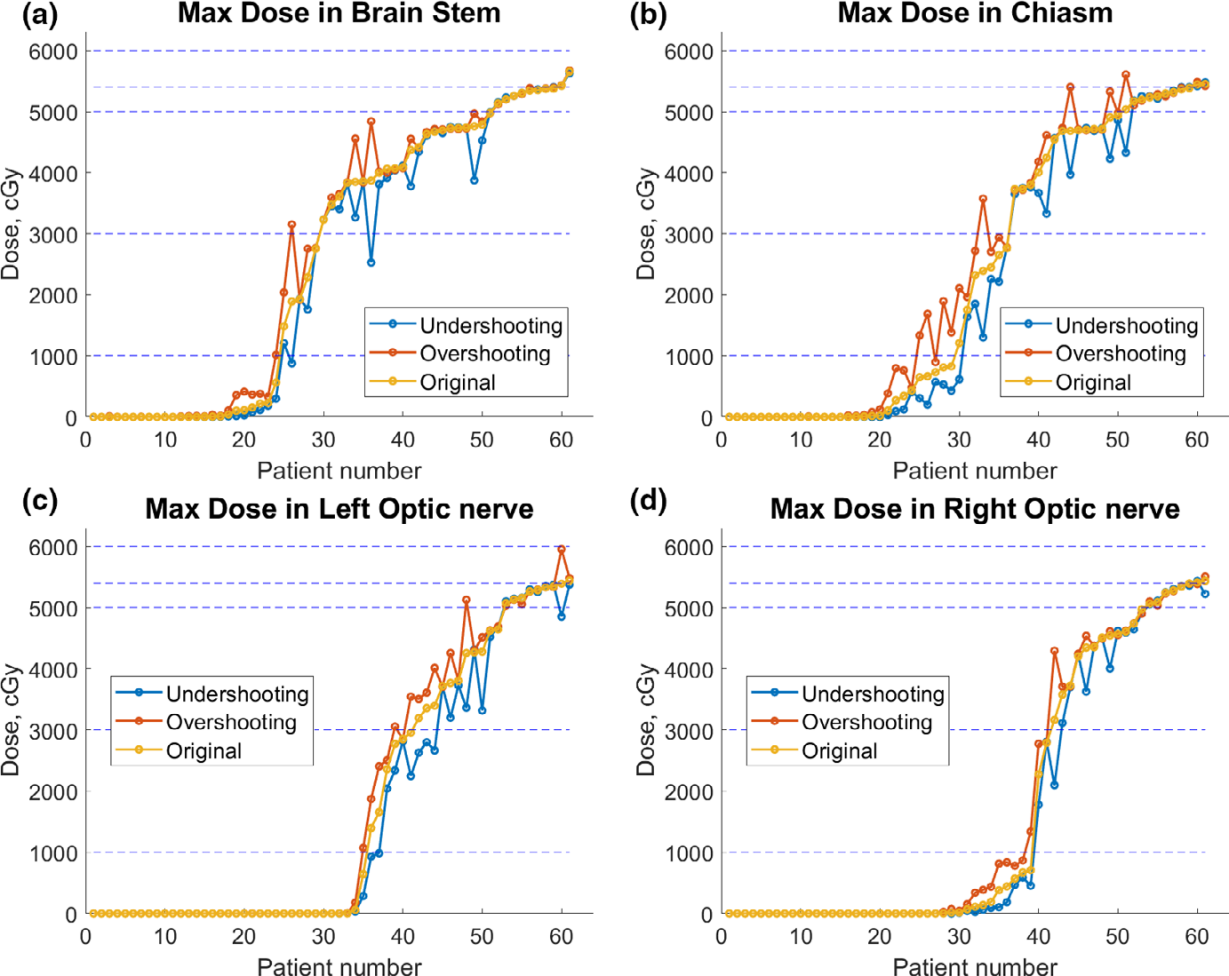
Distribution of deviations from planned values for maximum doses to OARs for brain tumor cases.

The aforementioned dose for all three scenarios for the 61 brain tumor cases are displayed in Fig. 1. As shown in Fig. 1(a), for brainstem, all overshooting scenarios consistently led to higher maximum dose than the corresponding original plans, while the undershooting scenarios behaved in an opposite way. However, even with overshooting by 3.5% in beam range, the maximum dose in the brainstem was <50 Gy in most of the cases, a good indicator that the range uncertainty is clinically accounted in general for brainstem toxicity, considering the prevailing dose limit for brainstem at 54 Gy. The jump in the maximum dose of OAR with 3.5% overshooting in range were mostly <5% but one case was about 25% from the corresponding original plan. Similar trends were observed for maximum doses in chiasm, left optic nerve and right optic nerve as shown in Figs. 1(b), 1(c), 1(d), respectively. After examining the maximum dose in OARs under the three scenarios and beam arrangement employed in clinical plans, range uncertainty impact seemed to be reduced successfully by means of margins or optimization of other planning parameters, including careful selection of beam angles and distal blocking, mainly due to the awareness of the risk with range uncertainty.

The percentage of minimum dose in CTV and CTV D95 with respect to prescription dose for all 61 brain cases under three scenarios are shown in the Fig. 1 of appendix. Overshooting scenarios had better minimum dose distribution in CTV compared to the original and undershooting scenarios. However, the improvement on minimum dose in CTV in the overshooting scenario was much less in extent than the deterioration in the undershooting scenario. The percentage of CTV D95 under three scenarios were very close to each other, which means that the overshooting and undershooting scenarios do not have substantial influence on the CTV D95.

**Fig. 2:**
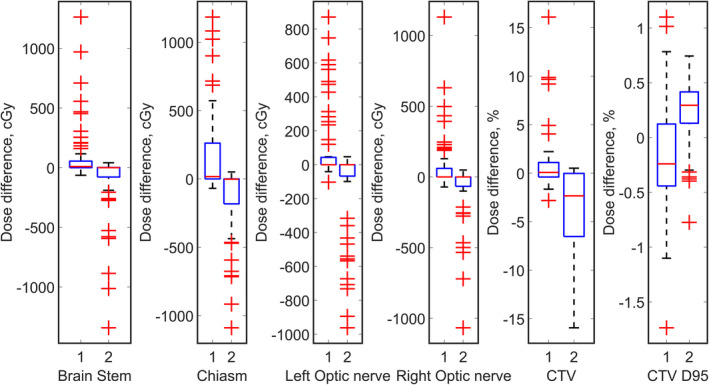
Boxplots of distribution of deviations from planned values for maximum doses to OARs, minimum dose to CTV, and D95 of CTV for brain tumor cases. The indices 1 and 2 represent overshooting plan and undershooting scenarios, respectively. The tops and bottoms of each box are the 25th and 75th percentiles of the samples, respectively. The distances between the tops and bottoms are the interquartile ranges. The line in the middle of each box is the sample median. The whiskers are lines extending above and below each box. Whiskers are drawn from the ends of the interquartile ranges to the furthest observations within the whisker length (the adjacent values). Observations beyond the whisker length are marked as outliers. The outlier is a value that is more than 1.5 times the interquartile range away from the top or bottom of the box. Outliers beyond the box were plotted in red markers.

For the 61 brain tumor cases, Fig. 2 shows that for most cases, differences between maximum OAR dose between the overshooting and undershooting scenarios and the original plans are small. However, for a few cases (marked as outliers in boxplots), the overshooting scenarios would lead to higher maximum dose of OARs, and the undershooting scenarios behaved in a reverse way.

**Fig. 3:**
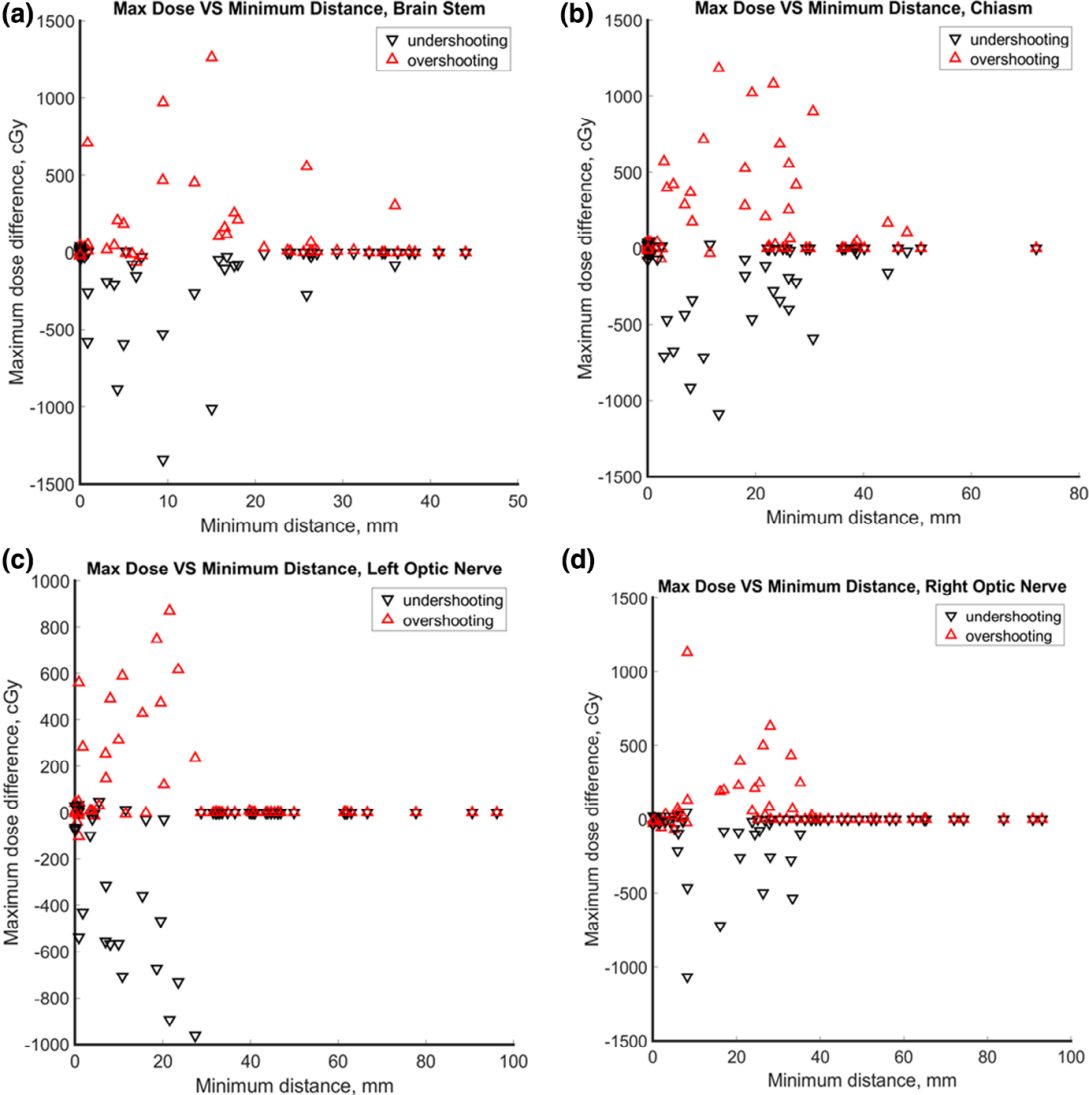
Maximum dose vs. minimum distance between OAR for brain tumor cases where the red and black triangles denote overshooting and undershooting scenarios, respectively.

The dependence of maximum OAR dose error on minimum CTV‐to‐OAR distance is shown in Fig. 3. Figure 3(a) shows large impact of range uncertainty on brainstem sparing for distances <20 mm from CTV. The impact tapered off with distance and was less than 5% for distances >30 mm. Correspondingly, a cut‐off distance was defined as the distance from an OAR to CTV beyond which impact from range uncertainty is considered negligible (<5%). Similar trends were observed in chiasm, left optic nerve and left optic nerve as illustrated in Figs. 3(b), 3(c), and 3(d), respectively. The cut‐off distances were about 30, 30, and 40 mm, for chiasm, left optic nerve and right optic nerve, respectively.

### 3.B Lung cancer cases

**Fig. 4:**
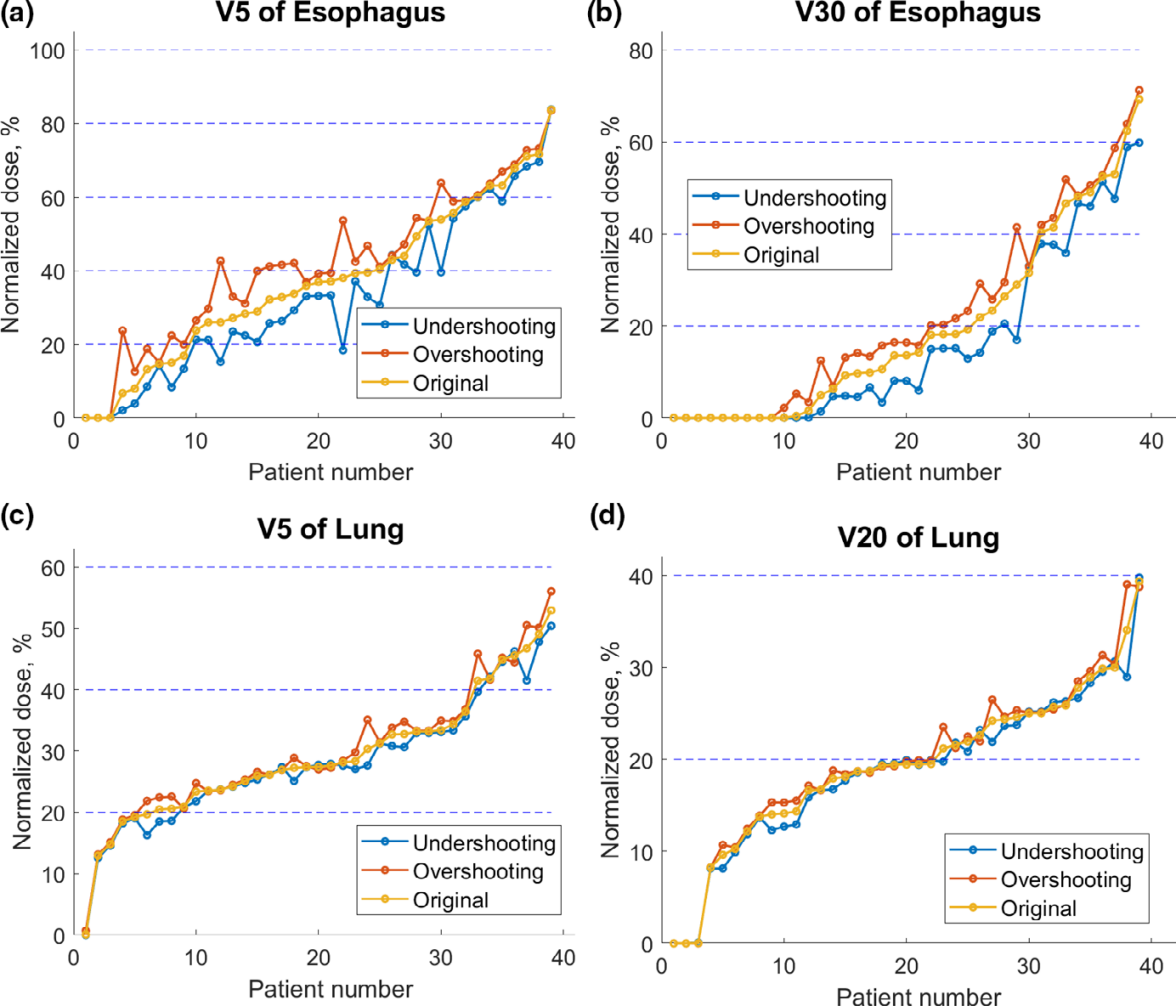
Dose distribution of lung and esophagus for lung tumor cases.

Figure 4 shows the deviations of OAR DVH metrics from their planned values for 39 lung tumor cases. For all endpoints, overshooting and undershooting scenarios consistently exhibit higher and lower doses, respectively, relative to the original plan. As shown in Fig. 4(a), for V5 Gy of esophagus, in all three plans, most of the dose volume coverage (coverage in cumulative dose volume histogram) was less than 80%, and one case had dose volume which was higher than 80%. The difference of dose volume of OAR under the plan with 3.5% range uncertainty and original plan could be over 5% but <10% for all the selected cases. A similar trend applied to V30 Gy of esophagus, V5 Gy of lung, V20 Gy of lung as shown in Figs. 4(b), 4(c), 4(d), respectively. The data for V5 Gy of heart, and V30 Gy of heart are shown in Fig. 2 of Appendix. The V100% of CTV is shown in Fig. 3 of Appendix. For most of the cases, the overshooting scenario leads to higher V100% of CTV.

**Fig. 5:**
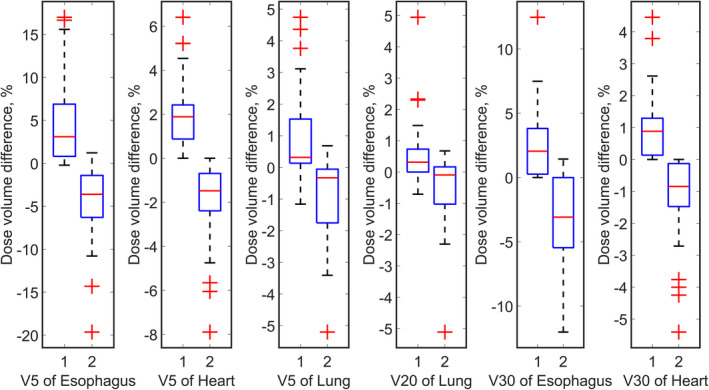
Boxplots of dose distribution of lung, heart, and esophagus for lung tumor cases. In the boxplots, id 1 and 2 represent overshooting scenario and undershooting scenario, respectively. The tops and bottoms of each box are the 25th and 75th percentiles of the samples, respectively. The distances between the tops and bottoms are the interquartile ranges. The line in the middle of each box is the sample median. The whiskers are lines extending above and below each box. Whiskers are drawn from the ends of the interquartile ranges to the furthest observations within the whisker length (the adjacent values). Observations beyond the whisker length are marked as outliers. The outlier is a value that is more than 1.5 times the interquartile range away from the top or bottom of the box. Outliers beyond the box were plotted in red markers.

Figure 5 shows the difference of dose volume of OARs between the overshooting scenarios and the original plans, and the difference of dose volume of OARs between the undershooting scenarios and the original plans. We observed that the differences of dose volume are small between the overshooting scenarios and the original plans, and same pattern applied to the undershooting scenarios. For a few cases, the overshooting scenarios would lead to higher dose volume of OARs, and the undershooting scenarios behaved in a reverse way.

**Fig. 6:**
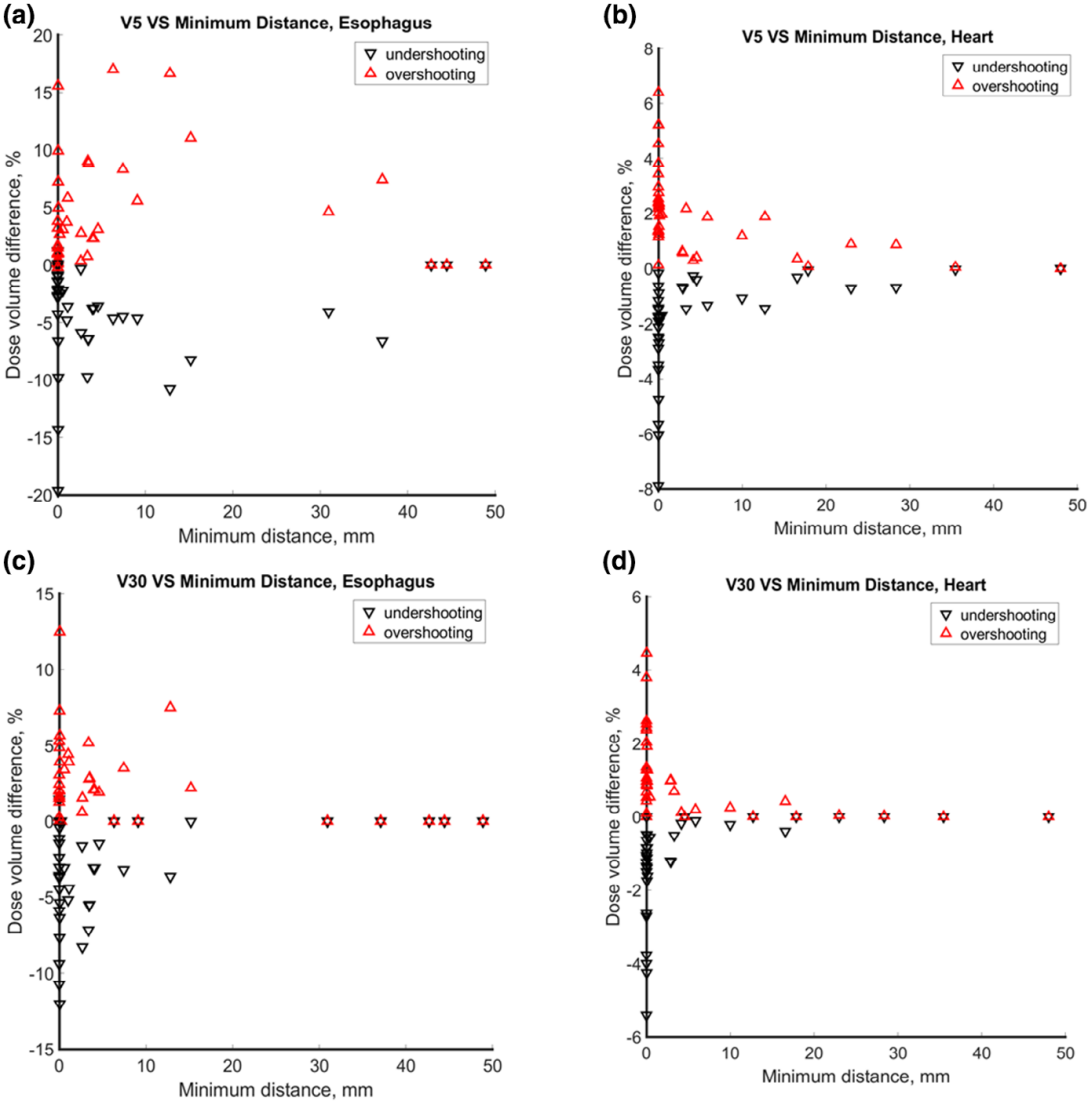
Dose volume vs. minimum distance between OAR and CTV for lung tumor cases. The red triangle indicates the dose by overshooting scenario, and the black triangle indicates the dose by undershooting scenario.

For lung tumor cases, the difference of dose volume of OAR under the plan with 3.5% range uncertainty and original plan with respect to the minimum distance between OAR and CTV is shown in Fig. 6. As shown in Fig. 6(a), the overshooting scenario and undershooting scenario had substantial impact on V5 Gy when the minimum distance below 40 mm. Overshooting scenario led to higher V5 Gy, while undershooting scenario led to lower V5 Gy. We also observed similar trend for V5 Gy of heart, V30 Gy of esophagus, and V30 Gy of heart as shown in Figs. 6(b), 6(c), and 6(d), respectively. The cut‐off distances for V5 Gy of heart, V30 Gy of esophagus, and V20 Gy heart were 30, 15, and 20 mm, respectively. Though esophagus and heart are relatively large critical organs, the uncertainties still could pose a big challenge for the organs that are inevitably located distal to incident proton beams.

### 3.C Prostate cases

**Fig. 7:**
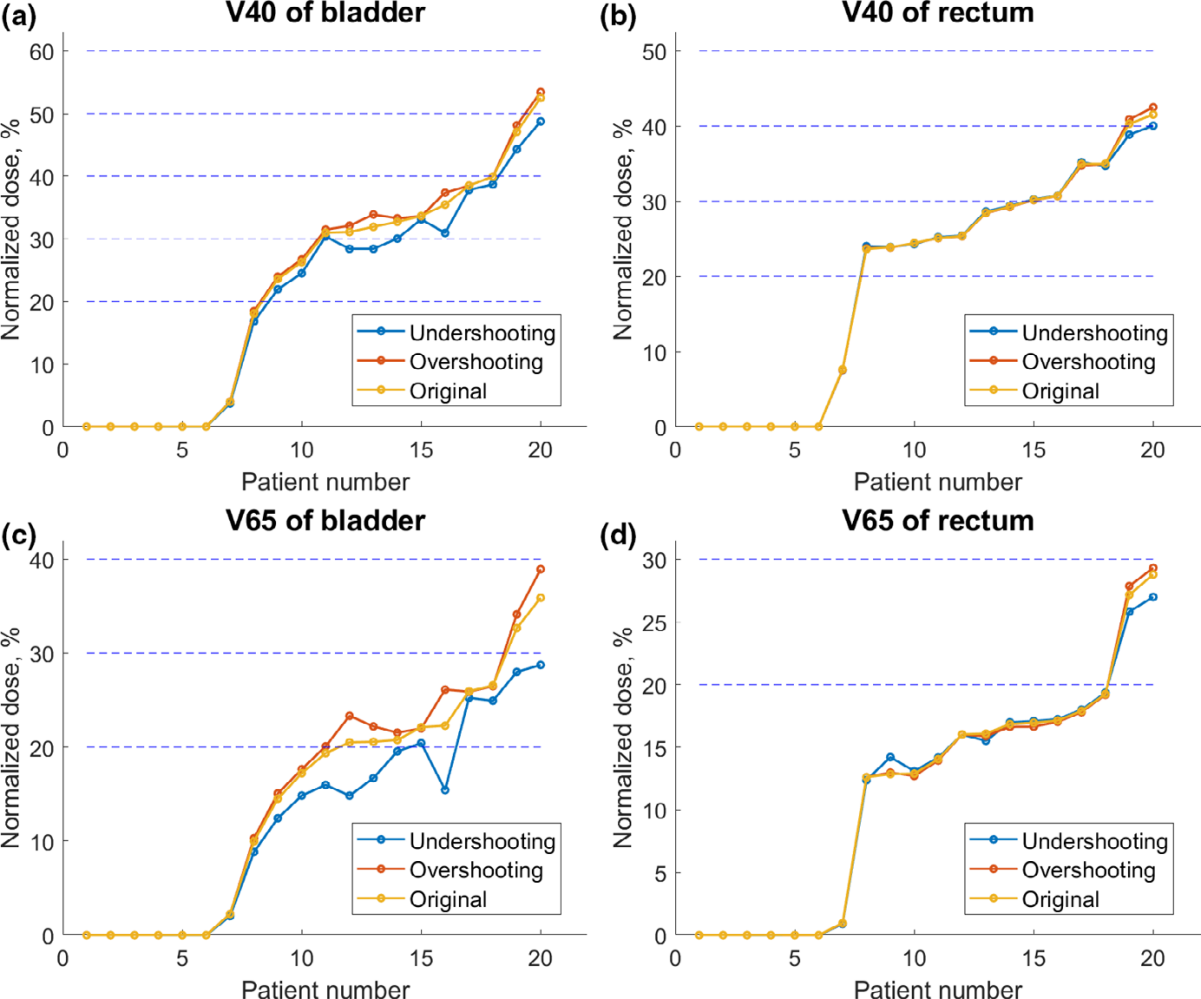
Dose distribution of bladder and rectum of prostate tumor cases.

The DVH metrics for bladder and rectum in the 20 prostate cases are shown in Fig. 7. We observed that overshooting scenario consistently leads to slightly higher dose volume than the original plan. As shown in Fig. 7(a), for the dose volume of V40 Gy of bladder, the undershooting scenario led to slightly lower dose volume than original plan. A similar trend applied to V40 Gy of rectum, V65 Gy of bladder, and V65 Gy of rectum as shown in Figs. 7(b), 7(c), 7(d), respectively. The V100% of CTV is shown in Fig. 4 of Appendix. For most of the cases, the overshooting scenario leads to higher V100% of CTV.

**Fig. 8:**
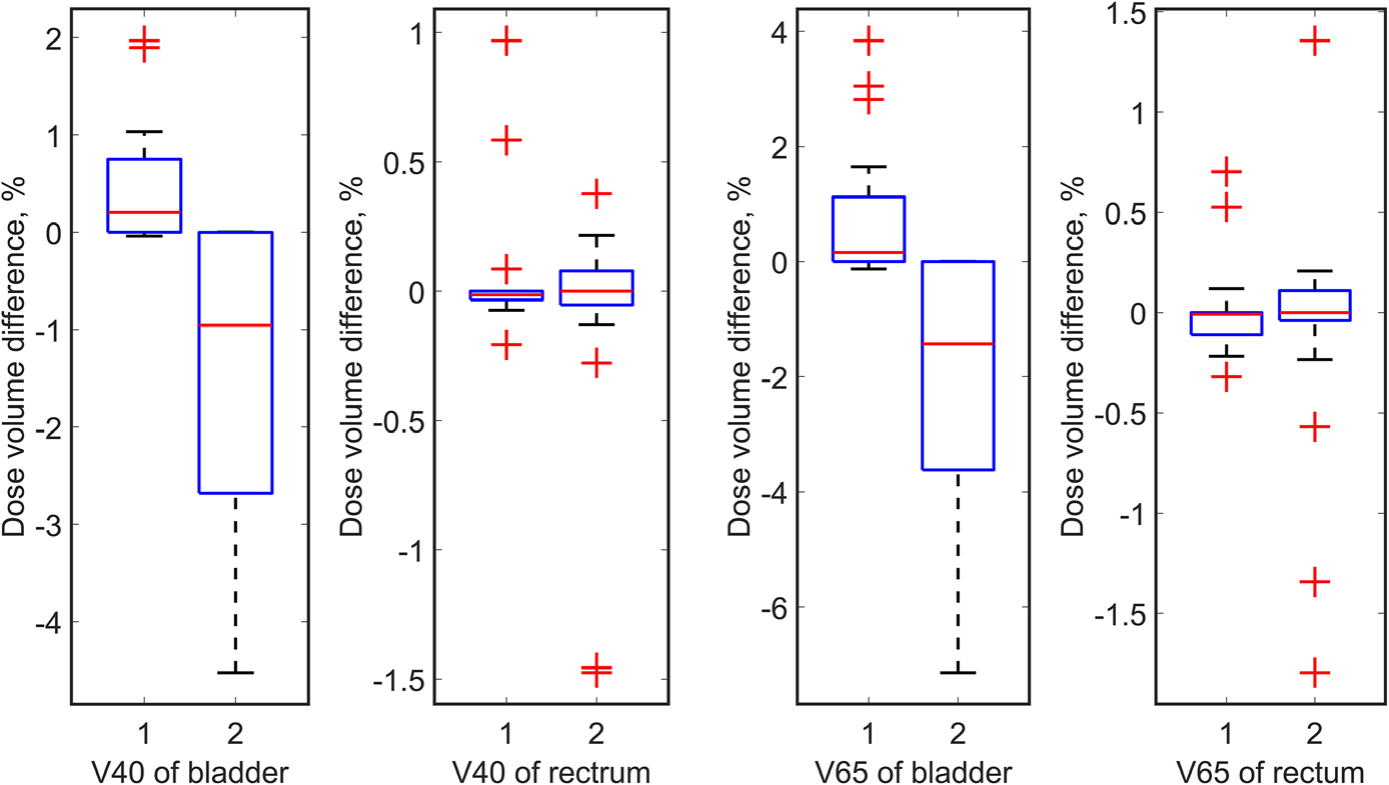
Dose distribution of bladder and rectum of prostate tumor cases. In the boxplots, id 1 and 2 represent overshooting scenario and undershooting scenario, respectively.

For the 20 prostate cancer cases, as shown in Fig. 8, the dose volume differences among the three plans are smaller than for brain tumor and lung cancer patients. This is because prostate cancer patients are treated with parallel opposed lateral fields, with bladder and rectum slightly overlapping the anterior and posterior margins of the treated volume, respectively, while range uncertainties mainly affect coverage of the right and left CTV and PTV margins. Only for very few cases, does the overshooting and undershooting scenarios led to 5% or larger changes in the bladder and rectal dose coverage.

**Fig. 9:**
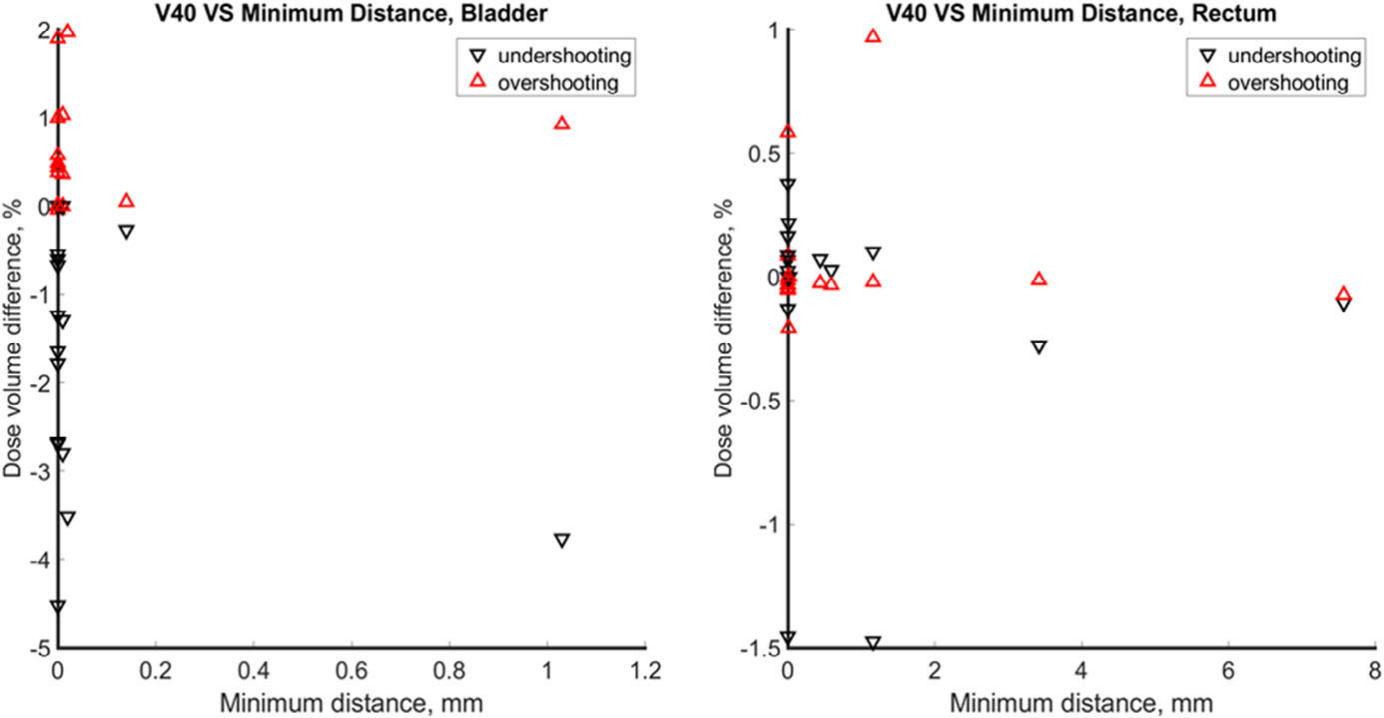
Dose volume vs minimum distance between OAR and CTV for prostate tumor cases.

For prostate tumor cases, the difference of dose volume of OAR under the plan with 3.5% range uncertainty and original plan with respect to the minimum distance between OAR and CTV is also shown in Fig. 9. The red triangle indicates the dose by overshooting scenario, and the black triangle indicates the dose by undershooting scenario As shown in Fig. 9(a), the overshooting scenario and undershooting scenario didn’t have substantial impact on V40 Gy of bladder regardless of their overlaps with or distances to the tumor, as demonstrated in Figs. 9(a) and 9(b). The uncertainties do not pose any significant challenges for bladder and rectum that are located lateral to incident proton beams.

## 4 DISCUSSION

Uncertainty in proton dose delivery arises from three major sources: temporal variations in anatomy, dose‐calculation algorithm limitations, and uncertainties in proton stopping power ratios used for dose calculation. Determining the relative impact of each source is beyond the scope of this study, which only focuses on the dosimetric impact from uncertainty in proton stopping power ratio.

Accurate acquisition of proton stopping power ratio clinically through traditional imaging process is impractical without a commercially available proton CT solution, although great progresses have been achieved in the reconstruction of proton stopping power with dual‐energy CT.^25^ The key holdback in the conversion from CT attenuation to proton stopping power is the mean excitation energy, which poses a ±1.5% uncertainty on proton range if no additional information of expect effective atomic number is included in an empirical equation for the mean excitation energy.^18^ To prioritize the coverage on tumor, additional margin is included in treatment plan to ensure that treatment target gets prescription dose in the case when reconstructed proton stopping power ratio from CT images is lower than the true value. However, this systematic addition to proton range would deliver unexpected extra dose to OARs around the tumor if reconstructed proton stopping power ratio is higher than the true value. Multiple measures have been taken to mitigate the risk of overshooting into OARs, including additional beams incident from different angles to avoid distal fall on the same OAR and uncertainty analysis. This retrospective study aims to evaluate the span of dosimetric impacts in tumor and OARs from ±3.5% range uncertainty for patients treated with passively scattered proton.

As a general result, the overshooting scenario delivered more dose to the OARs while the undershooting scenario reduced the dose to the OARs. The distal penumbra of proton beam is between 5–7 mm. For serial organs, which are typically in the brain, proton range is typically 15 cm. With 3% range uncertainty, the distal dose might move into the critical organ by about 5 mm. However, a few cases maintained a minimal distance between OARs and CTV beyond 5 mm. With careful selection of patients and optimizing the beam angles, we are able to minimize the impact of range uncertainty. For parallel organs, if the organ is lateral to CTV, the range uncertainty has very minimal impact. If the organ is distal to CTV, the impact of range uncertainty to the organ is mainly determined by the volume of the organ, compare lung with esophagus and heart. It needs further investigation if the treatment complications are caused by range uncertainty or uncertainty in biologically effective dose (BED).

The excessive dose is more severe in serial organs such as optic apparatus and brain stem where a single point dose dominated the toxicity, compared to parallel organs such as lung, heart and rectum, etc. The dose typically tapers off with distance, however, some impact of range uncertainty is still present at 4 cm from the surface of the tumor. The swings from the original plan narrow down in general as the DVH points in serial organs approach dose constraints, indicating that the strategies taken to mitigate the impact of range uncertainty actually work in the desired direction.

## 5 CONCLUSION

In conclusion, we investigated the dosimetric impact of range uncertainty of patients receiving passive scatter proton therapy retrospectively. Considering range uncertainties of ±3.5% for during treatment planning, the maximum dose to critical organs were maintained most brain patients, large variations over 5% were still observed sporadically. The Coverage in lung and prostate patients were less likely to be affected by range uncertainty. The margin recipe in modern TPS leads to clinically acceptable OAR doses in the presence of range uncertainties. However, range uncertainties still pose a noticeable challenge for small but critical serial organs near tumors, and occasionally for large parallel organs that are located distal to incident proton beams. Range uncertainty could cause unexpected dose to OARs. However, the risks can be minimized by careful selection of patients; optimizing beam angles to avoid the critical organ on the distal fall‐off of multiple beams.

## AUTHORS CONTRIBUTION

Ruirui Liu contributed to conceptualization, methodology, software, writing‐original draft, writing‐review and editing. Baozhou Sun contributed to review and editing. Tiezhi Zhang contributed to review and editing. Jeffery F. Williamson contributed to review and editing. Joseph A. O’Sullivan contributed to funding acquisition, review, and editing. Tianyu Zhao contributed to resources, conceptualization, methodology, supervision, writing, review, and editing.

## Supporting information


**Fig. S1**. The distribution of deviations from planned values for minimum dose to CTV, and D95 of PTV for brain tumor cases. The CTV dose and PTV dose were normalized to the prescription dose.

**Fig. S2**. Dose distribution of heart for lung tumor cases.

**Fig. S3**. The distribution of deviations from planned values for V100% of Lung for lung tumor cases.

**Fig. S4**. The distribution of deviations from planned values for V100% of prostate for prostate tumor cases.
