# Supplementary figures and images for: Dosimetric impact of range uncertainty in passive scattering proton therapy
Source: J Appl Clin Med Phys. 2021 Apr 2;22(5):6–14. doi: 10.1002/acm2.13179 (PMC8130244; doi:10.1002/acm2.13179)

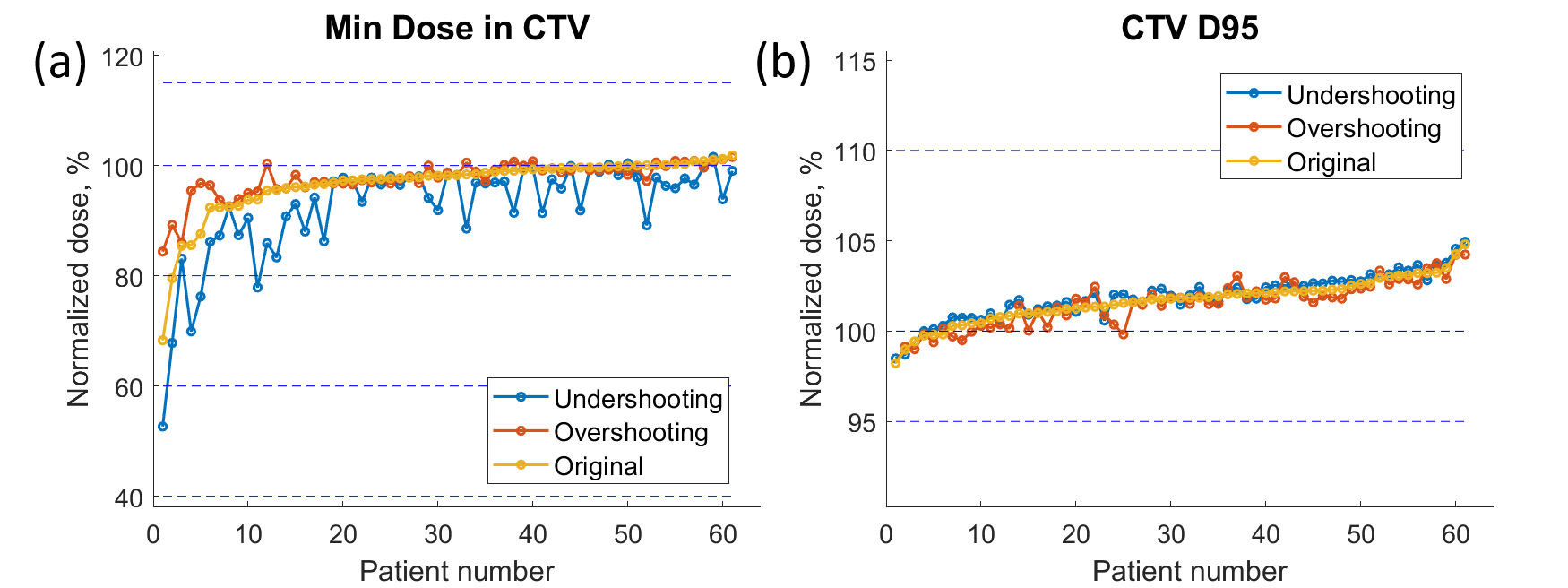

Supplement: Supplementary file 1 — Fig. S1. The distribution of deviations from planned values for minimum dose to CTV, and D95 of PTV for brain tumor cases. The CTV dose and PTV dose were normalized to the prescription dose. [file ACM2-22-6-s003.png]

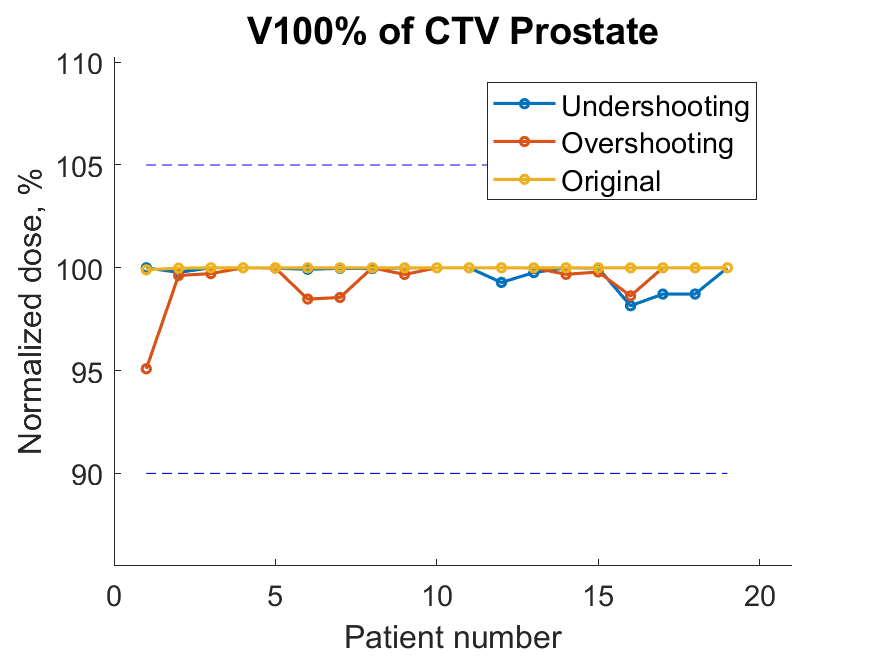

Supplement: Supplementary file 2 — Fig. S2. Dose distribution of heart for lung tumor cases. [file ACM2-22-6-s002.png]

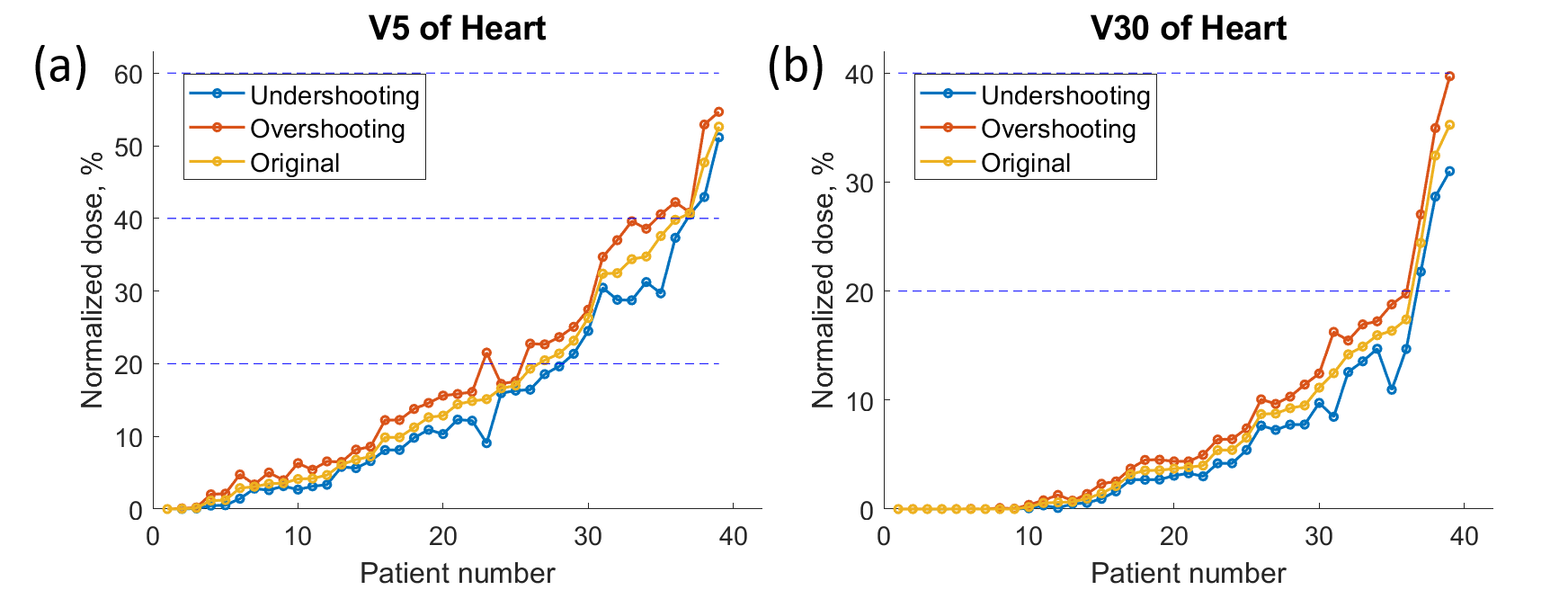

Supplement: Supplementary file 3 — Fig. S3. The distribution of deviations from planned values for V100% of Lung for lung tumor cases. [file ACM2-22-6-s001.png]

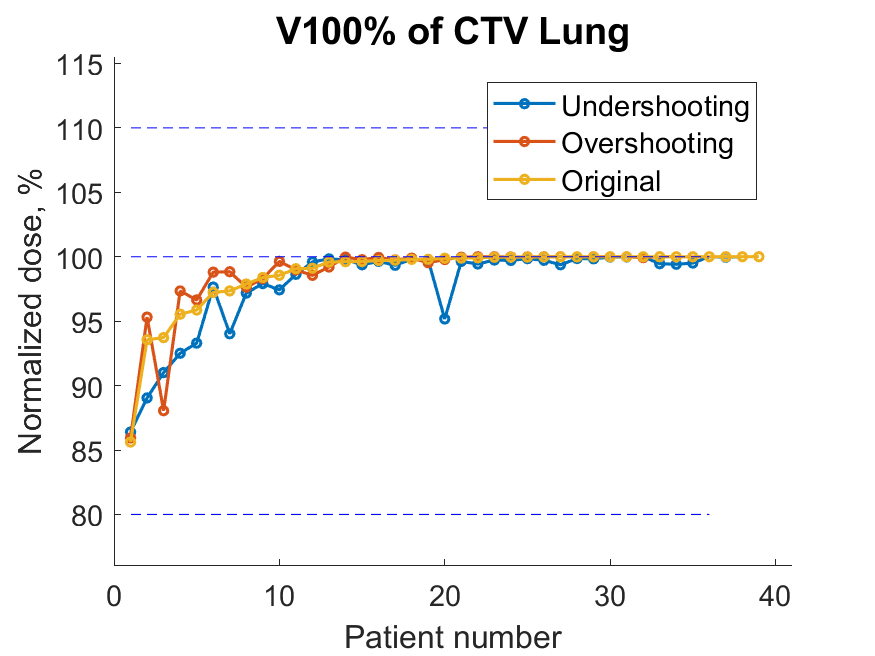

Supplement: Supplementary file 4 — Fig. S4. The distribution of deviations from planned values for V100% of prostate for prostate tumor cases. [file ACM2-22-6-s004.png]
